# *Notes from the Field:* Increase in Acute Hepatitis B Infections — Pasco County, Florida, 2011–2016

**DOI:** 10.15585/mmwr.mm6707a6

**Published:** 2018-02-23

**Authors:** Maura Comer, James Matthias, Garik Nicholson, Alice Asher, Scott Holmberg, Craig Wilson

**Affiliations:** ^1^Florida Department of Health, Bureau of Communicable Disease, STD and Viral Hepatitis Section, Tallahassee, Florida; ^2^Epidemiology and Statistics Branch, Division of STD Prevention, CDC; ^3^Florida Department of Health, Pasco County, Hudson, Florida; ^4^Epidemiology and Surveillance Branch, Division of Viral Hepatitis, CDC.

In April 2016, CDC noted an increase in acute hepatitis B virus (HBV) infections in Pasco County, Florida, through the National Notifiable Disease Surveillance System. Hepatitis B is an infection of the liver caused by HBV, which is transmitted through blood, semen, or other body fluids and is usually an acute, self-limiting illness in adults; however, some infected adults develop chronic HBV infection. HBV infection is preventable by vaccination. The Florida Department of Health (DOH-Florida) confirmed the local surveillance data; although Pasco County has fewer than half a million residents, in 2016, it had the highest number (87) and rate (17.28 per 100,000 population) of acute HBV infections among all Florida counties. From 2011 to 2016, the number of acute HBV-infected persons in Pasco County who met the national case definition* increased from 1.5 to 17.28 per 100,000 residents (p<0.001).

In mid-July 2016, DOH-Florida and Pasco County Department of Health (DOH-Pasco) epidemiologists initiated weekly conference calls to discuss strategies for preventing further infections within the county. Epidemiologic case surveillance data were reviewed to determine which risk factors were driving the increases in acute HBV infections. As of February 2017, among 275 cases of acute HBV infection reported in Pasco County during 2011–2016, risk factor information was ascertained for 221 (80%) patients. Among these, more than half (N = 113; 51%) reported some type of drug use, including 86 (39%) who reported injection drug use in the 6 months preceding symptom onset (Table) and 42 (19%) who reported incarceration for ≥24 hours during that time. Overall, 55% of reported HBV infections occurred in men and 45% in women. The observed increase in acute HBV infection related to injection drug use in Pasco County was similar to that seen in other Southern urban counties (*1*) and paralleled national trends in opioid use and overdose deaths (*2*).

**TABLE:** Demographic characteristics of persons with acute hepatitis B virus infection (N = 275) and reported risk factors — Pasco County, Florida, 2011–2016

Characteristic	No. (%)						
	2011	2012	2013	2014	2015	2016	Total (N = 275) (% of total)
	(n = 7)	(n = 25)	(n = 39)	(n = 53)	(n = 64)	(n = 87)	
**Race/Ethnicity**							
White, non-Hispanic	6 (86)	19 (76)	32 (82)	45 (85)	58 (91)	75 (86)	**235 (85)**
Black, non-Hispanic	0	1 (4)	0	0	0	1 (1)	**2 (1)**
Other, non-Hispanic	0	0	0	0	1 (2)	0	**1 (<1)**
Hispanic	1 (14)	0	1 (3)	0	2 (3)	3 (3)	**7 (3)**
Unknown	0	5 (20)	6 (15)	8 (15)	3 (4)	8 (9)	**30 (11)**
**Sex**							
Male	4 (57)	13 (52)	22 (56)	34 (64)	30 (47)	48 (55)	**151 (55)**
Female	3 (43)	12 (48)	17 (44)	19 (36)	34 (53)	39 (45)	**124 (45)**
**Age group (yrs)**							
19–29	1 (14)	1 (4)	2 (5)	2 (4)	2 (3)	8 (9)	16 (6)
30–39	2 (28.5)	10 (40)	18 (46)	19 (36)	24 (37.5)	26 (30)	**99 (36)**
40–49	2 (28.5)	7 (28)	12 (31)	15 (28)	18 (28)	29 (33)	**83 (30)**
50–59	2 (28.5)	5 (20)	4 (10)	11 (21)	14 (22)	12 (14)	**48 (17)**
60–69	0 (0)	2 (8)	3 (8)	4 (7.5)	5 (8)	9 (10)	**23 (8)**
≥70	0 (0)	0 (0)	0 (0))	2 (4)	1 (1.5)	3 (3)	**6 (2)**
Investigated*	**6 (86)**	**23 (92)**	**35 (90)**	**44 (90)**	**49 (77)**	**64 (74)**	**221 (80)**
**Risks** ^†^							
Any drug use	3 (50)	7 (30)	19 (54)	23 (52)	25 (51)	36 (56)	**113 (51)**
IDU^§^	3 (50)	5 (22)	14 (40)	18 (41)	21 (43)	25 (39)	**86 (39)**
Non-IDU^§^	2 (33)	3 (13)	9 (26)	17 (39)	20 (17)	32 (50)	**83 (38)**
Incarcerated >24 hours in last 6 months	2 (33)	4 (17)	6 (17)	4 (9)	7 (14)	19 (30)	**42 (19)**
Incarcerated >6 months in lifetime	2 (33)	1 (4)	5 (14)	12 (27)	9 (18)	13 (20)	**42 (19)**
Ever treated for an STI	2 (33)	6 (26)	3 (9)	5 (11)	13 (27)	5 (8)	**34 (15)**

**Abbreviations**: IDU = injection drug use; STI = sexually transmitted infection.

* Five or more of eight major risk factor questions answered.

^†^ Risk factor percentages calculated by dividing the number of persons with a given risk factor by the 221 investigated.

^§^ Might exceed total reporting any drug use because respondents might report both IDU and non-IDU history.

Since September 2016, DOH-Pasco epidemiology staff members have been collaborating with HIV and Sexually Transmitted Diseases program personnel and clinical staff to establish targeted outreach for testing and hepatitis B vaccination programs for persons at risk, including at a methadone clinic, at free health care clinics, and via a mobile medical unit operated by the Pasco County Public Defender’s Office. Law enforcement personnel helped to identify areas where drug users congregate, and DOH-Pasco worked with local jails and hospitals to identify and test persons who are at the highest risk for acquiring HBV infection. These efforts have resulted in administration of >300 hepatitis B vaccine doses in communities with persons at high risk for infection. One local hospital is now sending specimens to CDC for molecular characterization of HBV to delineate transmission networks in the county, using CDC’s Global Health, Outbreak, and Surveillance Technology (*3*). HBV surveillance data available for January–April 2017 indicated an 80% decrease in the number of acute cases of HBV infection compared with the same period in 2016. The decline likely represents a saturation of HBV among risk populations, the impact of hepatitis B vaccination and other interventions, or a combination of these factors. Pasco County is continuing enhanced HBV surveillance and prevention activities.

## Footnotes

* https://wwwn.cdc.gov/nndss/conditions/hepatitis-b-acute/case-definition/2012/.
